# The economic impact of a bioterrorist attack: are prevention and postattack intervention programs justifiable?

**DOI:** 10.3201/eid0302.970201

**Published:** 1997

**Authors:** A F Kaufmann, M I Meltzer, G P Schmid

**Affiliations:** Centers for Disease Control and Prevention, Atlanta, Georgia 30333, USA.

## Abstract

Understanding and quantifying the impact of a bioterrorist attack are essential in developing public health preparedness for such an attack. We constructed a model that compares the impact of three classic agents of biologic warfare (Bacillus anthracis, Brucella melitensis, and Francisella tularensis) when released as aerosols in the suburb of a major city. The model shows that the economic impact of a bioterrorist attack can range from an estimated $477.7 million per 100,000 persons exposed (brucellosis scenario) to $26.2 billion per 100,000 persons exposed (anthrax scenario). Rapid implementation of a postattack prophylaxis program is the single most important means of reducing these losses. By using an insurance analogy, our model provides economic justification for preparedness measures.


### The Economic Impact of a Bioterrorist Attack: Are Prevention and Postattack Intervention Programs Justifiable?

**Arnold F. Kaufmann, Martin I. Meltzer, and George P. Schmid**
Centers for Disease Control and Prevention, Atlanta, Georgia, USA

Understanding and quantifying the impact of a bioterrorist attack are essential in developing public health preparedness for such an attack. We constructed a model that compares the impact of three classic agents of biologic warfare (*Bacillus anthracis*, *Brucella melitensis*, and *Francisella tularensis*) when released as aerosols in the suburb of a major city. The model shows that the economic impact of a bioterrorist attack can range from an estimated $477.7 million per 100,000 persons exposed (brucellosis scenario) to $26.2 billion per 100,000 persons exposed (anthrax scenario). Rapid implementation of a postattack prophylaxis program is the single most important means of reducing these losses. By using an insurance analogy, our model provides economic justification for preparedness measures.

Bioterrorism and its potential for mass destruction have been subjects of increasing international concern. Approximately 17 countries (including five implicated as sponsors of international terrorism) may have active research and development programs for biologic weapons (1). Moreover, groups and individuals with grievances against the government or society have been known to use or plan to use biologic weapons to further personal causes.

Only modest microbiologic skills are needed to produce and effectively use biologic weapons. The greatest, but not insurmountable, hurdle in such an endeavor may be gaining access to a virulent strain of the desired agent. Production costs are low, and aerosol dispersal equipment from commercial sources can be adapted for biologic weapon dissemination. Bioterrorists operating in a civilian environment have relative freedom of movement, which could allow them to use freshly grown microbial suspensions (storage reduces viability and virulence). Moreover, bioterrorists may not be constrained by the need for precise targeting or predictable results.

The impact of a bioterrorist attack depends on the specific agent or toxin used, the method and efficiency of dispersal, the population exposed, the level of immunity in the population, the availability of effective postexposure and/or therapeutic regimens, and the potential for secondary transmission. Understanding and quantifying the impact of a bioterrorist attack are essential to developing an effective response. Therefore, we have analyzed the comparative impact of three classic biologic warfare agents (*Bacillus anthracis*, *Brucella melitensis*, and *Francisella tularensis*) when released as aerosols in the suburbs of a major city and compared the benefits of systematic intervention with the costs of increased disease incidence (from the economic point of view used in society).

### Analytic Approach

### Scenario Assumptions

We compared the impact of a theoretical bioterrorist attack on a suburb of a major city, with 100,000 population exposed in the target area. The attack was made by generating an aerosol of an agent (*B. anthracis* spores, *B. melitensis*, or *F. tularensis*) along a line across the direction of the prevailing wind. The meteorologic conditions (thermal stability, relative humidity, wind direction and speed) were assumed to be optimal (2), and the aerosol cloud passed over the

Address for correspondence: Martin I. Meltzer, Mail Stop C-12, National Center for Infectious Diseases, Centers for Disease Control and Prevention, Atlanta, GA 30333; fax: 404-639-3039; e-mail: qzm4@cdc.gov.

target area within 2 hours. We projected impact on the basis of 10% and 100% of the target population being exposed to the aerosol cloud.

We assumed that, when inhaled, the infectious $dose_{50}$ ($ID_{50}$) was 20,000 spores for *B. anthracis* and 1,000 vegetative cells for *B. melitensis* and *F. tularensis*. The rate of physical decay for airborne particles 5 µm or less in diameter was estimated to be negligible during the 2-hour transit time. The rate of biologic decay of the particulate agents was estimated to be negligible for the *B. anthracis* spores and 2% per minute for the *B. melitensis* and *F. tularensis* vegetative cells. Viability and virulence did not dissociate. Persons who were exposed to the *B. anthracis* cloud at any point during the 2-hour transit time inhaled one $ID_{50}$ dose, and persons who were exposed to either the *B. melitensis* or *F. tularensis* cloud inhaled one to 10 $ID_{50}$ doses, depending on their proximity to the origination point of the aerosol cloud.

The epidemic curve for anthrax by days after exposure was assumed to be <1 day, 0% of cases; 1 day, 5%; 2 days, 20%; 3 days, 35%; 4 days, 20%; 5 days, 10%; 6 days, 5%; and 7 or more days, 5% (3-5). Case-fatality rates were also assumed to vary by the day symptoms were first noted. The case-fatality rate was estimated as 85% for patients with symptoms on day 1; 80% for patients with symptoms on day 2; 70% for those with symptoms on day 3; 50% for those with symptoms on days 4, 5, and 6; and 70% for those with symptoms on and after day 7. The increased death rate in persons with an incubation period of 7 or more days is calculated on an assumption of delayed diagnosis, with resultant delayed therapy.

When estimating days in hospital and outpatient visits due to infection, we assumed that 95% of anthrax patients were hospitalized, with a mean stay of 7 days. Patients not admitted to a hospital had an average of seven outpatient visits, and surviving hospitalized patients had two outpatient visits after discharge from the hospital. Persons who received only outpatient care were treated for 28 days with either oral ciprofloxacin or doxycycline. No significant long-term sequelae resulted from the primary infection, and no relapses occurred.

The epidemic curve for brucellosis by days after exposure was assumed to be 0 to 7 days, 4% of cases; 8 to 14 days, 6%; 15 to 28 days, 14%; 29 to 56 days, 40%; 57 to 112 days, 26%, and 113 or more days, 10% (4, 6-9). The case-fatality rate was estimated to be 0.5%. Fifty percent of patients were hospitalized, with an average stay of 7 days. Nonhospitalized patients had an average of 14 outpatient visits, and hospitalized patients had seven outpatient visits after discharge from the hospital. Outpatients received a combination of oral doxycycline for 42 days and parenteral gentamicin for the first 7 days of therapy. Five percent of patients had a relapse or long-term sequelae, and required 14 outpatient visits within 1 year.

The epidemic curve for tularemia by days after exposure was assumed to be: <1 day, 0% of cases; 1 day, 1%; 2 days, 15%; 3 days, 45%; 4 days, 25%; 5 days, 10%; 6 days, 3%; and 7 or more days, 1% (4,10-11). The estimated case-fatality rate was 7.5%; and 95% of patients were hospitalized, with an average stay of 10 days. Nonhospitalized patients had an average of 12 outpatient visits, and hospitalized patients who survived the acute illness had two outpatient visits after discharge from the hospital. Outpatients received oral doxycycline for 14 days and parenteral gentamicin for 7 days. Five percent of patients had a relapse or long-term sequelae and required an average of 12 outpatient visits.

The efficacy of intervention strategies is unknown; our projections are our best estimates based on published clinical and experimental data (4,12-14). For anthrax, the projected intervention program was either a 28-day course of oral ciprofloxacin or doxycycline (assumed to be 90% effective), or a 28-day course of oral ciprofloxacin or doxycycline plus three doses of the human anthrax vaccine (assumed to be 95% effective); for brucellosis, a 42-day course of oral doxycycline and rifampin (assumed to be 80% effective), or a 42-day course of oral doxycycline, plus 7 days of parenteral gentamicin (assumed to be 95% effective); for tularemia, the intervention program was a 14-day course of oral doxycycline (assumed to be 80% effective), or a 14-day course of oral doxycycline plus 7 days of parenteral gentamicin (assumed to be 95% effective). Only 90% of persons exposed in the target area were assumed to effectively participate in any intervention program. Because the target area cannot be precisely defined, we estimated that for every exposed person participating in the intervention program, an additional 5, 10, or 15 nonexposed persons would also participate.

### Economic Analyses of Postattack Intervention

To analyze the economic factors involved in establishing an intervention program, we compared the costs to the potential savings from such an intervention. Following the recommendation of the Panel of Cost-Effectiveness in Health and Medicine (PCEHM), we used estimates of actual costs rather than financial charges or market prices, which usually incorporate profit (15). We calculated the net savings (cost reductions) by using the following formula: Net savings = (number of deaths averted x present value of expected future earnings) + (number of days of hospitalization averted x cost of hospitalization) + (number of outpatient visits averted x cost of outpatient visits) - cost of intervention.

When we calculated the costs of hospitalization and outpatient visits, we assumed that only persons with symptoms (i.e., case-patients) would use medical facilities. The remainder of the exposed and potentially exposed populace would receive postexposure prophylaxis.

### Present Value of Expected Future Earnings

The cost of a premature human death was nominally valued at the present value of expected future earnings and housekeeping services, weighted by the age and sex composition of the work force in the United States (16). The undiscounted average of future earnings is $1,688,595. As recommended by PCEHM (17), the stream of future earnings was discounted at 3% and 5%, to give values of $790,440 and $544,160, respectively. The present value of expected future earnings was estimated with 1990 dollars, adjusted for a 1% annual growth in productivity (16). However, in constant terms (1982 dollars), the average hourly earnings in private industry fell from $7.52 in 1990 to $7.40 in 1994 (18); therefore, the estimate of future earnings was not adjusted upwards.

### Cost of Hospitalization

In 1993, the average charge for a single day of hospitalization was $875 (19). To derive true cost, we multiplied the average charge by the cost-to-charge ratio of 0.635, (the April 1994 statewide average cost-to-charge ratio for urban hospitals in New York state) (16). On this basis, we estimated true hospitalization costs at $556/day (Table 1). Hospital costs included all professional services, drugs, x-rays, and laboratory tests. Lost productivity during hospital stay was valued at $65/day (the value of an “unspecified” day’s earnings, weighted for age and sex composition of the U.S. work force) (16).

### Cost of Posthospitalization Outpatient Visits

After discharge from the hospital, a patient was assumed to have follow-up outpatient visits, the number of which varied by disease (Table 1). Outpatient visit costs were valued by using the Medicare National Average Allowance (20), which was chosen to represent the equivalent of bulk purchase discounted costs (i.e., actual costs) (Table 1). The first visit has a Current Procedural Terminology (CPT) code of 99201, which is classified as a “level 1” visit, requiring a physician to spend an average of 10 minutes with a patient (20). Subsequent level 1 visits, with the physician spending an average of 5 minutes with each patient, have a CPT code of 99211 (20). During outpatient visits, a general health panel test incorporating clinical chemistry tests and complete blood counts (CPT code 80050) and a single antigen or antibody detection test (e.g., CPT code 86558) were assumed to be ordered (20). Although data on Medicare allowances for office visits and many other procedures were available, data on Medicare allowances for laboratory tests were not. Thus, to establish the costs of the tests, we arbitrarily divided the lowest allowable charge for each test in half. X-rays (CPT code 71021) were valued according to the Medicare National Average Allowance (Table 1). In terms of lost productivity, we assumed that each outpatient visit cost the equivalent of 2 hours, or one-quarter, of the value of an unspecified day (16).

### Cost of Outpatient Visits of Nonhospitalized Patients

For nonhospitalized outpatients, the cost of each visit, laboratory test, x-ray, and lost productivity was the same as an outpatient visit for discharged hospital patients and varied by disease (Table 1). We assumed that one set of laboratory tests would be ordered every other visit and that two sets of x-rays (CPT code 71021) would be ordered during the therapeutic course. Drug costs are discussed below.

### Cost of an Intervention

The costs of an intervention can be expressed as follows: Cost of intervention = (cost of drugs used) x ([number of people exposed x multiplication

Table 1. Costs of hospitalization and outpatient visits (OPVs) following a bioterrorist attack

| | Anthrax | | Tularemia | | Brucellosis | |
|---|---|---|---|---|---|---|
| | Base | Upper | Base | Upper | Base | Upper |
| *Hospitalized patient* | | | | | | |
| Days in hospital | 7 | 7 | 10 | 10 | 7 | 7 |
| Cost per day ($)[a] | 556 | 669 | 556 | 669 | 556 | 669 |
| Lost productivity ($/day) | 65 | 65 | 65 | 65 | 65 | 65 |
| Follow-up OPVs (no.) | 2 | 2 | 2 | 2 | 7 | 7 |
| Cost 1st OPV ($) | 28 | 44 | 28 | 44 | 28 | 44 |
| Cost other OPVs, ea. ($) | 13 | 24 | 13 | 24 | 13 | 24 |
| OPV laboratory ($)[b,c] | 87 | 174 | 87 | 174 | 131 | 261 |
| OPV x-rays costs ($)[d] | 66 | 66 | 0 | 0 | 0 | 0 |
| Lost productivity ($/OPV)[e] | 16 | 16 | 16 | 16 | 16 | 16 |
| Total costs ($) | 4,541 | 5,380 | 6,338 | 7,582 | 4,584 | 5,587 |
| Avg. costs/day ($/day) | 649 | 769 | 634 | 758 | 655 | 798 |
| *% increase: Base to upper estimate* | | *18* | | *20* | | *22* |
| *Nonhospitalized patient* | | | | | | |
| Number of OPVs | 7 | 7 | 12 | 12 | 14 | 14 |
| Cost 1st OPV ($) | 28 | 44 | 28 | 44 | 28 | 44 |
| Cost other OPVs, ea. ($) | 13 | 24 | 13 | 24 | 13 | 24 |
| Lost productivity ($/OPV)[e] | 16 | 16 | 16 | 16 | 16 | 16 |
| Laboratory costs ($)[b,f] | 131 | 174 | 261 | 522 | 261 | 522 |
| X-ray costs ($)[d] | 66 | 66 | 66 | 66 | 66 | 66 |
| Drugs used[g] | D | C | D+G | D+G | D+R | D+R+G |
| Cost of drugs ($) | 6 | 181 | 29 | 29 | 220 | 246 |
| Total costs ($) | 422 | 810 | 722 | 1,120 | 972 | 1,418 |
| Avg. costs/day ($/day) | 60 | 116 | 60 | 93 | 69 | 101 |
| *% increase: Base to upper estimate* | | *93* | | *55* | | *46* |

Notes: All costs rounded to the nearest whole dollar.
[a]Hospital costs assumed to include all costs such as drugs, laboratory tests, and x-rays.
[b]Laboratory tests consists of general health panel (CPT code 80050) and an antigen or antibody test (modeled on the cost of a *Streptococcus* screen, CPT code 86588).
[c]Follow-up OPVs for hospitalized patients included two laboratory test sets for anthrax and tularemia patients and three laboratory test sets for brucellosis patients.
[d]X-ray costs (CPT code 71021), included two sets taken at different OPVs.
[e]Productivity lost due to an OPV was assumed to be one-quarter of an unspecified day's value.
[f]For OPVs of nonhospitalized patients, one set of laboratory tests is assumed for every two visits.
[g]Drugs used: D = doxycycline; C = ciprofloxacin; R = rifampin.
Sources: See text for explanation of sources of cost estimates.

factor] - number killed - number hospitalized - number of persons who require outpatient visits).

The intervention costs per person depend directly on the costs of the antimicrobial agents and vaccines used in a prophylaxis program (Table 2). We obtained drug prices from the 1996 Drug Topics Red Book and used the lowest cost available for each drug (21). The cost of doxycycline ($0.22 per 200 mg total daily dose) was the Health Care Financing Administration cost, whereas the cost of gentamicin ($3.76 per 160 mg total daily dose), ciprofloxacin ($3.70 per 1,000 mg total daily dose), and rifampin ($5.01 per 900 mg total daily dose) were wholesale costs from pharmaceutical companies. The cost of anthrax vaccine was $3.70 per dose (Helen Miller-Scott, pers. comm., 1996). The cost of administering one vaccine dose or gentamicin injection was estimated at $10.00, on the basis of the 1992 cost of administering a vaccine in a clinical setting (Valerie Kokor, pers. comm., 1996). In estimating the cost of administering oral antimicrobial agents, we assumed weekly visits, during which the drug would be distributed and counseling would be given ($15.00 for the first visit and $10.00 for each subsequent visit).

We assumed that more people would receive prophylaxis than were actually exposed because of general anxiety and uncertainty about the boundaries of the attack, the timing of the attack, and the time it would take nonresidents to travel through the attack area. Three different multiplication factors (5, 10, and 15) were used to construct

Table 2. Costs of prophylaxis following a bioterrorist attack

| Level of effectiveness | Anthrax | Tularemia | Brucellosis |
|---|---|---|---|
| *Lower* | | | |
| Effectiveness (%) | 90 | 80 | 80 |
| Drugs used[a] | D or C | D | D+R |
| Cost of drugs ($)[b] | 6 or 181 | 3 | 220 |
| No. of visits[c] | 4 | 2 | 6 |
| Total cost/ person ($) | 51 or 226 | 28 | 285 |
| *Upper* | | | |
| Effectiveness (%) | 95 | 95 | 95 |
| Drugs used[a] | D+V or C+V | D+G | D+G |
| Cost of drugs ($)[b] | 17 or 193 | 29 | 36 |
| No. of visits[c] | 4 | 7 | 12 |
| Total cost/ person ($) | 62 or 238 | 104 | 161 |
| Minimum No. participants[d] | 451,912 | 418,094 | 423,440 |
| Maximum No. participants[e] | 1,492,750 | 1,488,037 | 1,488,037 |

Notes: All costs are rounded to the nearest whole dollar.
[a]Drugs used: D = doxycycline; C = ciprofloxacin; V = anthrax vaccine; G = gentamicin; R = rifampin.
[b]See text for explanation of drug costs.
[c]Cost of visit to drug-dispensing site: 1st visit = $15/person; follow-up visits = $10/person/visit.
[d]Estimate assumed that the prophylaxis program was initiated on postattack day 6 for anthrax and tularemia and postattack day 113 for brucellosis, that the prophylaxis program had the lower effectiveness level, and that the multiplication factor for unnecessary prophylaxis givn to unexposed persons was 5.
[e]Estimate assumed that prophylaxis was initiated on postattack day 0 (day of release), that prophylaxis had the upper effectiveness level, and that the multiplication factor for unnecessary prophylaxis given to unexposed persons was 15.

alternative cost-of-intervention scenarios that take into account persons who were not at risk but participated in the prophylaxis program. Thus, if 100,000 people were exposed, we assumed that the maximum number seeking prophylaxis was 500,000, 1,000,000, or 1,500,000.

### Economic Analysis of Preparedness: Insurance

The analyses outlined above consider only the economics of an intervention after an attack and include several assumptions: First, stockpiles of drugs, vaccines, and other medical supplies would be available and could be rapidly moved to points of need. Second, civil, military, and other organizations would be in place and have the capability to rapidly identify the agent, dispense drugs, treat patients, and keep order within the population. Finally, ongoing intelligence gathering would detect possible bioterrorist threats. The cost of these prerequisite activities can be calculated if they are seen as a form of insurance, the goal of which is to "purchase" the maximum net savings through preparedness to manage the consequences of an attack and reduce the probability of an attack. The "actuarially fair premium" for the "insurance" can be defined as follows (22): Actuarially fair premium = reduction of loss probability x value of avoidable loss.

The term "reduction of loss probability" indicates that, although increased surveillance and related activities can reduce the odds of an attack, they cannot guarantee absolute protection. The term "avoidable loss" refers to the fact that, even if a postexposure prophylaxis program were implemented on the day of release (day zero), some deaths, hospitalizations, and outpatient visits would be unavoidable.

Various reductions of attack probability illustrated the impact of these estimates on the calculation of actuarially fair premiums. Such reductions included reducing the probability from 1 in 100 years (0.01) to 1 in 1,000 years (0.001), a reduction of 0.009, and reducing a probability from 1 in a 100 years (0.01) to 1 in 10,000 years (0.0001), and from 1 in 100 years (0.01) to 1 in 100,000 years (0.00001). The attack probability of 0.01 in the absence of enhanced preventive actions was selected for illustrative purposes and does not represent an official estimate.

A range of minimum and maximum values of avoidable loss was derived from the net savings calculations. The values reflect differences in effectiveness of the various prophylaxis regimens, the reduced impact of delayed prophylaxis on illness and death, and the two discount rates used to calculate the present value of earnings lost because of death.

### Sensitivity Analyses

In addition to the scenarios discussed above, three sensitivity analyses were conducted. First, the impact of increasing the cost of hospitalization and outpatient visits was assessed by using a set of upper estimates (Table 1). The cost of a hospital day was increased to $669 by increasing the cost-to-charge ratio from 0.634 to 0.764 (the ratio for Maryland) (16). The costs of outpatient visits (first and follow-up) were increased by assuming each visit was a "level 2" visit, doubling the average time a physician spends with each patient. The costs of laboratory tests were increased to the full amount of the allowable charge (20).

The second sensitivity analysis considered a reduced impact, in which only 10% of the original 100,000 target population were considered exposed. All other estimates were held constant. The third sensitivity analysis considered the threshold cost of an intervention, given differences due to the effectiveness of various drug regimens, and discount rates used to calculate the present value of expected lifetime earnings lost to a death. The threshold cost occurs when net savings equal $0. Thus, the threshold value represents the maximum that could be spent per person on an intervention without having the intervention cost more than the loss from no intervention.

### Findings

### Postattack Illness and Death

In our model, all three biologic agents would cause high rates of illness and death. In the absence of an intervention program for the 100,000 persons exposed, the *B. anthracis* cloud would result in 50,000 cases of inhalation anthrax, with 32,875 deaths; the *F. tularensis* cloud in 82,500 cases of pneumonic or typhoidal tularemia, with 6,188 deaths; and the *B. melitensis* cloud in 82,500 cases of brucellosis requiring extended therapy, with 413 deaths.

The speed with which a postattack intervention program can be effectively implemented is critical to its success (Figure 1). For diseases with short incubation periods such as anthrax and tularemia, a prophylaxis program must be instituted within 72 hours of exposure to prevent the maximum number of deaths, hospital days, and outpatient visits (Figure 1). Some benefit, however, can be obtained even if prophylaxis is begun as late as day 6 after exposure. The relative clinical efficacy of the intervention regimen has a lesser but definite impact on observed illness and death rates (Figure 1).

A disease with a long incubation period such as brucellosis has a similar pattern (Figure 1); an important difference is the time available to implement an intervention program. Having more time available to implement an intervention program can make a marked difference in its effectiveness. However, the prolonged incubation period creates a greater potential for panic in potentially exposed persons because of the uncertainty about their health status.

### Economic Analyses of Postattack Intervention: No Program

Without a postexposure prophylaxis program, an attack with *B. anthracis* is far costlier than attacks with *F. tularensis* or *B. melitensis* (Table 3). The differences between agents in medical costs as a percentage of total estimated costs are due to the large differences in death rates attributed to each agent (Figure 1).

### Net Savings Due to a Postexposure Prophylaxis Program

If the postexposure prophylaxis program is initiated early, it reduces the economic impact of all three diseases, especially anthrax (Figure 2). Regardless of drug costs, the largest cost reductions

Table 3. Costs[a] ($ millions) of a bioterrorist attack with no postexposure prophylaxis program

| | Anthrax | Tularemia | Brucellosis |
|---|---|---|---|
| *Direct costs* | | | |
| Medical: Base estimates[b] | | | |
| Hospital | 194.1 | 445.8 | 170.3 |
| OPV[c] | 2.0 | 10.5 | 48.9 |
| Medical: Upper estimates[d] | | | |
| Hospital | 237.1 | 543.3 | 211.7 |
| OPV[c] | 4.4 | 18.5 | 78.3 |
| *Lost productivity* | | | |
| Illness[e] | | | |
| Hospital | 21.6 | 50.9 | 18.8 |
| OPV[c] | 0.7 | 3.9 | 15.0 |
| Death | | | |
| 3% discount[f] | 25,985.7 | 4,891.2 | 326.5 |
| 5% discount[f] | 17,889.3 | 3,367.3 | 224.7 |
| *Total costs* | | | |
| Base estimates | | | |
| 3% discount[f] | 26,204.1 | 5,402.4 | 579.4 |
| 5% discount[f] | 18,107.7 | 3,878.4 | 477.7 |
| Upper estimates | | | |
| 3% discount[f] | 26,249.7 | 5,507.9 | 650.1 |
| 5% discount[f] | 18,153.1 | 3,983.9 | 548.4 |

[a]Assuming 100,000 exposed.
[b]Medical costs are the costs of hospitalization (which include follow-up outpatient visits) and outpatient visits (Table 1).
[c]OPV = outpatient visits.
[d]Upper estimates calculated with data in Table 1.
[e]Lost productivity due to illness is the value of time spent in hospital and during OPVs (Table 1).
[f]Discount rate applied to calculate the present value of expected future earnings and housekeeping services, weighted by age and sex composition of the United States workforce (16), lost due to premature death.

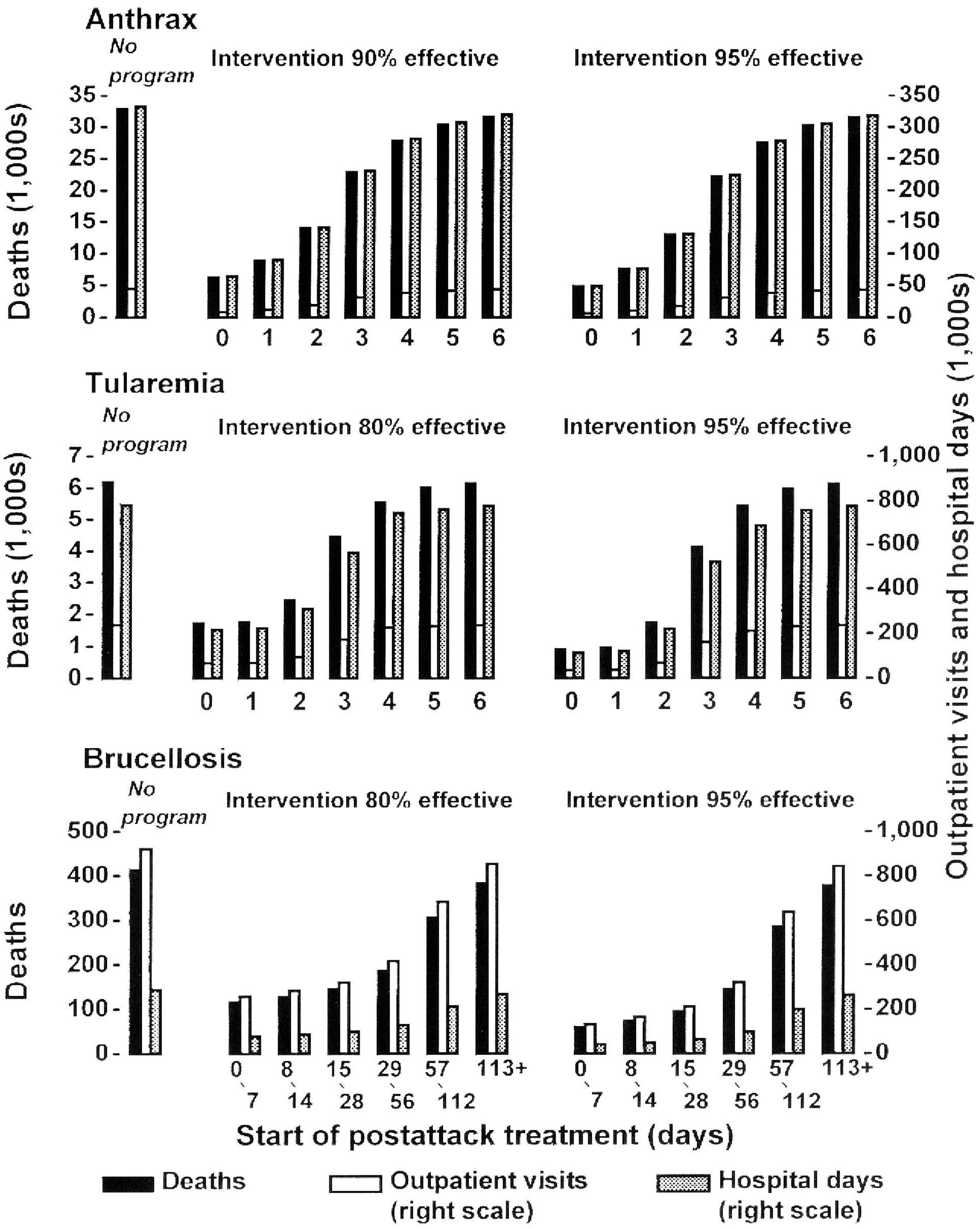

Figure 1. Total deaths, hospital days, and outpatient visits associated with aerosol releases of *B. anthracis*, *B. melitensis*, and *F. tularensis* by the postattack day of prophylaxis initiation and level of prophylaxis effectiveness.

are obtained through a combination of the most effective prophylaxis regimen (i.e., 95% effective, Table 2), the smallest multiplication factor to adjust for persons who unnecessarily receive prophylaxis, and a 3% discount rate to calculate the present value of the expected value of lifetime earnings.

In the case of anthrax, either doxycycline or ciprofloxacin could be used in the intervention program (Table 2), but the use of doxycycline generated the largest savings. The largest difference in net savings between the two drugs was approximately $261.6 million. This difference occurred when it was assumed that the program began on day zero (day of release), each drug was used in combination with the anthrax vaccine, a 3% discount rate was used, and a multiplication factor of 15 for unnecessary prophylaxis was used. This amount is equal to approximately 1.2% of the maximum total net savings generated by using a regimen of doxycycline plus the anthrax vaccine.

Some scenarios, particularly those in which prophylaxis programs were started late, generated negative net savings (i.e., net losses). In the case of tularemia, at a 5% discount rate, net losses of $10.7 to $115.1 million occurred when a post-exposure program was delayed until day 6 after exposure, and a prophylaxis regimen of doxycycline and gentamicin (estimated 95% efficacy) was used. For the same scenario, but with a 3% discount, a net savings of $1,513.3 million was observed when a multiplication factor of five for unnecessary prophylaxis was used. However, multiplication factors of 10 and 15 generated net losses of $49.8 and $102.0 million, respectively. With the same drug combination, beginning the program 1 day earlier (day 5 after exposure) resulted in net savings in all scenarios except when a multiplication factor of 15 and a discount rate of 5% were used. Under the latter two assumptions, net savings result only for prophylaxis initiated by day 4 after exposure.

In the case of brucellosis, the use of a doxycycline-rifampin regimen (estimated 80% efficacy), a multiplication factor of 15 for unnecessary prophylaxis, and a discount rate of either 3% or 5% generated net losses regardless of when intervention began (Figure 2). The doxycycline-gentamicin regimen (estimated 95% efficacy) generated net losses only when it was assumed that the start of a program was delayed until 113 or more days after exposure.

### Preparedness: Insurance

The annual actuarially fair premium that can be justifiably spent on intelligence gathering and other attack prevention measures increases with the probability that a bioterrorist attack can be decreased by such measures (Table 4). However, the potential net savings attributed to reduced probability are minor compared with the potential net savings from implementing a prophylaxis program. Depending on the level of protection that can be achieved, the annual actuarially fair pre-

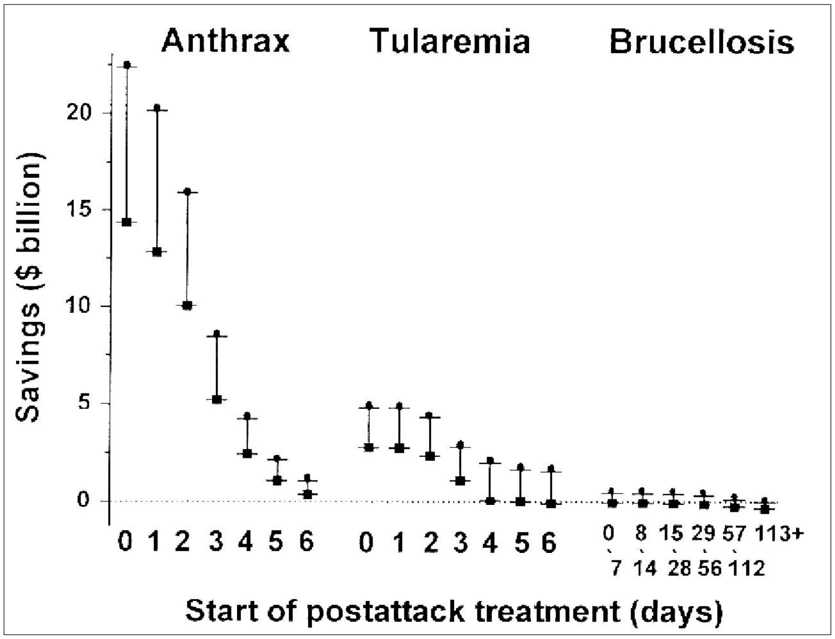

Figure 2. Ranges[a] of net savings due to postattack prophylaxis by disease and day of prophylaxis program initiation.

[a]Maximum savings (●) were calculated by assuming a 95% effectiveness prophylaxis regimen and a 3% discount rate in determining the present value of expected lifetime earnings lost due to premature death (16) and a multiplication factor of 5 to adjust for unnecessary prophylaxis. Minimum savings (■) were calculated by assuming an 80% to 90% effectiveness regimen and a 5% discount rate and a multiplication factor of 15. In tularemia prophylaxis programs initiated on days 4-7 postattack, the minimum savings were calculated by assuming a 95% prophylaxis regimen effectiveness rather than an effectiveness of 80% to 90%.

mium in an anthrax scenario would be $3.2 million to $223.5 million (Table 4). The lower premium would be justifiable for measures that could reduce the risk for an attack from 0.01 to 0.001 and provide the ability to mount an intervention program within 6 days of the attack. The higher premium would be justifiable for measures that could reduce the risk from 0.01 to 0.00001 and allow immediate intervention if an attack occurred.

### Sensitivity Analyses

The upper estimates of the cost of hospitalization increased average costs per day by 18% to 22%, and upper estimates of the cost of outpatient visits increased average costs per day by 46% to 93% (Table 1). However, the upper estimates only increased medical costs by 1% to 6% of the total medical costs associated with a bioterrorist attack (Table 3). The largest increase was for brucellosis, for which upper estimates increased medical costs from 38% to 44% of total costs (Table 3).

Table 4. The maximum annual actuarially fair premium[a] by reduction in probability of event and size of avoided loss: Anthrax

| Days post-attack[b] | Preventable loss ($millions) | Actuarially fair annual premium ($ millions) 0.01 to 0.001 | 0.01 to 0.0001 | 0.01 to 0.00001 |
|---|---|---|---|---|
| *Maximum loss estimate[c]* | | | | |
| 0 | 22,370.5 | 201.3 | 221.5 | 223.5 |
| 1 | 20,129.4 | 181.2 | 199.3 | 201.1 |
| 2 | 15,881.5 | 142.9 | 157.2 | 158.7 |
| 3 | 8,448.0 | 76.0 | 83.6 | 84.4 |
| 4 | 4,200.1 | 37.8 | 41.6 | 42.0 |
| 5 | 2,076.1 | 18.7 | 20.6 | 20.7 |
| 6 | 1,013.8 | 9.1 | 10.0 | 10.1 |
| *Minimum loss estimate[d]* | | | | |
| 0 | 14,372.4 | 128.9 | 141.8 | 143.1 |
| 1 | 12,820.1 | 115.4 | 126.9 | 128.1 |
| 2 | 10,049.1 | 90.4 | 99.5 | 100.4 |
| 3 | 5,200.1 | 46.8 | 51.5 | 51.9 |
| 4 | 2,429.7 | 21.9 | 24.1 | 24.3 |
| 5 | 1,004.2 | 9.4 | 10.3 | 10.4 |
| 6 | 351.2 | 3.2 | 3.5 | 3.5 |

[a]See text for definition.
[b]No. of days from attack to effective initiation of prophylaxis.
[c]Maximum loss preventable (potential net savings) occurs with the doxycycline-anthrax vaccine prophylaxis regimen, a multiplication factor of 5 for unnecessary prophylaxis, and a discount rate of 3% (Table 2).
[d]Minimum loss preventable (potential net savings) occurs with the ciprofloxacin prophylaxis regimen, a multiplication factor of 15 for unnecessary prophylaxis, and a discount rate of 5% (Table 2).

When the number of persons infected during an attack was reduced tenfold, the patient-related costs were reduced proportionately (Table 3). In most cases, however, the net savings in total costs are less than 10% of the net savings when 100% of the target population was presumed infected. The shortfall in savings is caused by an increase in the number of unexposed persons receiving prophylaxis. In the case of anthrax, when intervention programs are initiated within 3 days of exposure, savings are 4.1% to 10% of those in the original scenario (Figure 2). Delaying initiation of prophylaxis until days 4, 5, or 6 after exposure, however, results in net losses of $13.4 to $283.1 million. Losses occur regardless of prophylaxis regimen, discount rate, or multiplication factor used to adjust for unnecessary prophylaxis by unexposed persons.

In scenarios in which a multiplication factor of 15 was used to adjust for unnecessary prophylaxis, the threshold value of intervention was always above the prophylaxis cost for anthrax but not above the prophylaxis costs for tularemia and brucellosis (Table 5). For tularemia, the threshold intervention costs exceeded disease costs up to day 5 in the scenario with 95% effectiveness and a 5% discount, and for brucellosis, at all levels in the scenarios with 80% effectiveness and up to day 56 in the scenarios with 95% effectiveness. This is consistent with the lower range of estimated net savings (net losses) given in Figure 2. Reducing the number of unexposed persons receiving prophylaxis increases the cost thresholds, making the program cost beneficial. For example, changing the multiplication factors for unnecessary prophylaxis to 5 and 10 increases the cost thresholds to $659 and $319, respectively, for a brucellosis prophylaxis program initiated 15 to 28 days after exposure, with a 5% discount rate. If a discount rate of 3% is used instead of 5%, the cost thresholds increase to $799 and $387. All these cost thresholds are above the estimated prophylaxis cost of $285 per person for the doxycycline-rifampin regimen and $161 per person for the doxycycline-gentamicin regimen (Table 2).

### Conclusions

The economic impact of a bioterrorist attack can range from $477.7 million per 100,000 persons exposed in the brucellosis scenario to $26.2 billion per 100,000 persons exposed in the anthrax scenario (Table 3). These are minimum

Table 5. Cost thresholds[a] of interventions ($/person) by day of intervention initiation, prophylaxis effectiveness, and discount rates.

| Threshold costs for intervention ($/person, multiplication factor of 15[b]) | | | | | | | | |
|---|---|---|---|---|---|---|---|---|
| Anthrax | | | Tularemia | | | Brucellosis | | |
| Post-attack day[d] | Disc. rate[c] 5% | 3% | Post-attack day | Disc. rate 5% | 3% | Post-attack day | Disc. rate 5% | 3% |
| *90% effectiveness[e]* | | | *80% effectiveness[e]* | | | *80% effectiveness[e]* | | |
| 0 | 9,838 | 14,238 | 0 | 1,891 | 2,633 | 0-7 | 233* | 282* |
| 1 | 8,851 | 12,809 | 1 | 1,873 | 2,609 | 8-14 | 224* | 272* |
| 2 | 7,022 | 10,162 | 2 | 1,599 | 2,227 | 15-28 | 211* | 255* |
| 3 | 3,775 | 5,463 | 3 | 756 | 1,053 | 29-56 | 179* | 217* |
| 4 | 1,893 | 2,739 | 4 | 258 | 366 | 57-112 | 86* | 104* |
| 5 | 944 | 1,366 | 5 | 79 | 110 | 113+ | 24* | 30* |
| 6 | 468 | 677 | 6 | 20* | 28 | | | |
| *Prophylaxis cost[c]* | | *$226* | | | *$28* | | | *$285* |
| *95% effectiveness[e]* | | | *95% effectiveness[e]* | | | *95% effectiveness[e]* | | |
| 0 | 10,370 | 15,007 | 0 | 2,229 | 3,104 | 0-7 | 274 | 333 |
| 1 | 9,359 | 13,544 | 1 | 2,207 | 3,074 | 8-14 | 264 | 320 |
| 2 | 7,427 | 10,948 | 2 | 1,898 | 2,644 | 15-28 | 248 | 301 |
| 3 | 3,995 | 5,782 | 3 | 898 | 1,251 | 29-56 | 211 | 256 |
| 4 | 2,004 | 2,900 | 4 | 328 | 457 | 57-112 | 102* | 124* |
| 5 | 1,000 | 1,447 | 5 | 93* | 131 | 113+ | 29* | 35* |
| 6 | 496 | 718 | 6 | 23* | 32* | | | |
| *Prophylaxis cost[e]* | | *$238* | | | *$104* | | | *$161* |

*Threshold value is below estimated cost of prophylaxis.
[a]Cost threshold is the point where cost of intervention and net savings due to the intervention are equal.
[b]Multiplication factor to adjust for persons who participated in the prophylaxis program but were unexposed.
[c]Applied to present value of expected future earnings and housekeeping services (weighted average for age and sex).
[d]Postattack day on which prophylaxis was effecively implemented.
[e]See Table 2 for prophylaxis regimens assumed to give the stated levels of effectiveness and cost/person of prophylaxis.

estimates. In our analyses, we consistently used low estimates for all factors directly affecting costs. The $ID_{50}$ estimates for the three agents are twofold to 50-fold higher than previously published estimates (5,6,10,11), resulting in a possible understatement of attack rates. Also, in our analyses we did not include a number of other factors (e.g., long-term human illness or animal illnesses) (Table 6) whose cumulative effect would likely increase the economic impact of an attack.

Our model shows that early implementation of a prophylaxis program after an attack is essential. Although the savings achieved by initiating a prophylaxis program on any given day after exposure has a wide range, a clear trend of markedly reduced savings is associated with delay in starting prophylaxis (Figure 2). This trend was found in the analysis of all three agents studied.

Delay in starting a prophylaxis program is the single most important factor for increased losses (reduced net savings). This observation was supported by the actuarially fair premium for preparedness analysis (Table 4). Reductions in preventable loss due to early intervention had significantly greater impact on the amount of an actuarially fair premium than reductions in probability of an attack through intelligence gathering and related activities.

Although implemented at different times in a threat-attack continuum, both attack prevention measures and prophylaxis programs are forms of preventive medicine. Attack prevention measures seek to prevent infection, while prophylaxis programs prevent disease after infection has occurred.

Using an actuarially fair premium analogy in which cost and benefit are required to be equal, we find that the incremental rate of increasing prevention effectiveness (the marginal increase) declines rapidly as probability reduction targets go from 0.001 to 0.0001 to 0.00001. Because the loss probability is decreasing on a logarithmic scale, the potential increment in marginal benefit drops comparably, resulting in ever smaller increments in the protection above the preceding base level.

Conversely, delaying a prophylaxis program for anthrax, a disease with a short incubation

Table 6. Potential factors affecting the economic impact of a bioterrorist attack

| Factor | Potential impact on net savings | Relative magnitude of impact |
|---|---|---|
| Higher than projected case-fatality rate | Increase | ++++ |
| Long term illness (physical and psychological) | Increase | ++ |
| Decontamination and disposal of biohazardous waste | Increase | ++ |
| Disruptions in commerce (local, national, and international) | Increase | ++ |
| Animal illness and death | Increase | + |
| Lower than projected effectiveness of prophylaxis | Decrease | - - - |
| Adverse drug reactions due to prophylaxis | Decrease | - |
| Postattack prophylaxis distribution costs, including crowd control and security | Decrease | - |
| Training and other skill maintenance costs | Decrease | - |
| Procurement and storage of antimicrobial drugs and vaccines before attack | Decrease | - |
| Criminal investigations and court costs | Variable | +/- |

period and a high death rate, increases the risk for loss in a manner akin to a semilogarithmic scale. Arithmetic increases in response time buy disproportionate increases in benefit (prevented losses.) The potential for reducing loss is great because an attack is assumed, thus increasing the actuarially fair premium available to prepare for and implement a rapid response.

Large differences between prophylaxis costs and the threshold costs for most scenarios, particularly if prophylaxis is early (Table 5), suggest that the estimates of savings from prophylaxis programs are robust. Even with large increases in prophylaxis cost, net savings would still be achieved.

The ability to rapidly identify persons at risk would also have significant impact on costs. For example, the threshold costs for brucellosis prophylaxis are often lower than intervention costs when the ratio of unexposed to exposed persons in the prophylaxis program is 15:1 (Table 5). This finding provides an economic rationale for preparedness to rapidly and accurately identify the population at risk and reduce unnecessary prophylaxis costs.

The maximum amount of the annual actuarially fair premium varies directly with the level of risk reduction and the rapidity of postattack response (Table 4). The calculated amount of actuarially fair premiums, however, should be considered a lower bound estimate. A higher estimate (called the certainty equivalent) can also be calculated; however, this requires the determination of a social welfare function (22), and such complexity is beyond the scope of this study.

Our model provides an economic rationale for preparedness measures to both reduce the probability of an attack and increase the capability to rapidly respond in the event of an attack. The larger portion of this preparedness budget (insurance premium) should be allocated to measures that enhance rapid response to an attack. These measures would include developing and maintaining laboratory capabilities for both clinical diagnostic testing and environmental sampling, developing and maintaining drug stockpiles, and developing and practicing response plans at the local level. These measures should be developed with a value-added approach. For example, the laboratory capability could be used for other public health activities in addition to preparedness, and drugs nearing their potency expiration date could be used in government-funded health care programs. However, these secondary uses should not undermine the preparedness program's effectiveness.

Arnold Kaufmann is a retired Public Health Service officer, formerly assigned to the National Center for Infectious Diseases.

### References

1. Cole LA. The specter of biological weapons. Sci Am 1996;275:60-5.
2. Gochenour WS. Aerobiology. Mil Med 1963;128:86-9.
3. Abramova FAN, Grinberg LM, Yampolskaya OV, Walker DH. Pathology of inhalational anthrax in 42 cases from the Sverdlovsk outbreak of 1979. Proc Natl Acad Sci 1993;90:2291-4.
4. Benenson AS, editor. Control of communicable diseases manual. 16th ed. Washington (DC): American Public Health Association, 1995.
5. Messelson M, Guillemin J, Hugh-Jones M, Langmuir A, Popova I, Shelokov A, et al. The Sverdlosvsk anthrax outbreak of 1979. Science 1994;266:1202-8.
6. Kaufmann AF, Fox MD, Boyce JM, Anderson DC, Potter ME, Martone WJ, et al. Airborne spread of brucellosis. Ann NY Acad Sci 1980;335:105-14.
7. Olle-Goig JE, Canela-Soler J. An outbreak of *Brucella melitensis* infection by airborne transmission among laboratory workers. Am J Public Health 1987;77:335-8.

8. Staszkiewicz J, Lewis CM, Colville J, Zervos M, Band J. Outbreak of *Brucella melitensis* among microbiology laboratory workers in a community hospital. J Clin Microbiol 1991;29:287-90.
9. Trever RW, Cluff LE, Peeler RN, Bennett IL. Brucellosis I. laboratory-acquired acute infection. Arch Intern Med 1959;103:381-97.
10. McCrumb FR. Aerosol infection of man with *Pasteurella tularensis*. Bacteriolical Reviews 1961;25:262-7.
11. Saslaw S, Eigelsbach HT, Wilson HR, Prior JA, Carhart S. Tularemia vaccine study II. respiratory challenge. Arch Intern Med 1961;107:689-701.
12. Friedlander AM, Welkos SL, Pitt MLM, Ezzell JW, Worsham PL, Rose, KJ, et al. Postexposure prophylaxis against experimental inhalation anthrax. J Infect Dis 1993;167:1239-42.
13. Sawyer WD, Dangerfield HG, Hogge AL, Crozier D. Antibiotic prophylaxis and therapy of airborne tularemia. Bacteriolical Reviews 1966;30:542-8.
14. Solera J, Rodriguez-Zapata M, Geijo P, Largo J, Paulino J, Saez L, et al. Doxycycline-rifampin versus doxycycline-streptomycin in treatment of human brucellosis due to *Brucella melitensis*. Antimicrob Agents Chemother 1995;39:2061-7.
15. Luce BR, Manning WG, Siegel JE, Lipscomb J. Estimating costs in cost-effectiveness analysis. In: Gold MR, Siegel JE, Russell LB, Weinstein MC, editors. Cost-effectiveness in health and medicine. New York: Oxford University Press, 1966:176-213.
16. Haddix AC, Teutsch SM, Shaffer PA, Dunet DO, editors. Prevention effectiveness: a guide to decision analysis and economic evaluation. New York: Oxford University Press, 1996.
17. Lipscomb J, Weinstein MC, Torrance GW. Time preference. In: Gold MR, Siegel JE, Russell LB, Weinstein MC, editors. Cost-effectiveness in health and medicine. New York: Oxford University Press, 1966:214-35.
18. U.S. Bureau of the Census. Statistical abstract of the United States: 1995. 115th ed. Washington (DC): U.S. Government Printing Office, 1996.
19. National Center for Health Statistics. Health, United States, 1995. Hyattsville (MD): U.S. Department of Health and Human Services, Public Health Service, 1996.
20. HealthCare Consultants of America, Inc. HealthCare Consultants' 1996 physicians fee and coding guide. 6th ed. Augusta (GA): HealthCare Consultants of America, Inc. 1996.
21. Cardinale V, editor. 1996 Drug Topics Red Book. Montvale (NJ): Medical Economics Company, Inc., 1996.
22. Robison LJ, Barry PJ. The competitive firm's response to risk. New York: Macmillan, 1987.
